# Intranasal Immunization with Recombinant HA and Mast Cell Activator C48/80 Elicits Protective Immunity against 2009 Pandemic H1N1 Influenza in Mice

**DOI:** 10.1371/journal.pone.0019863

**Published:** 2011-05-20

**Authors:** Shu Meng, Zhonghua Liu, Lili Xu, Li Li, Shan Mei, Linlin Bao, Wei Deng, Lina Li, Rongyue Lei, Liangzhi Xie, Chuan Qin, Linqi Zhang

**Affiliations:** 1 AIDS Research Center, Institute of Pathogen Biology, Chinese Academy of Medical Sciences and Peking Union Medical College, Beijing, China; 2 Institute of Laboratory Animal Sciences, Chinese Academy of Medical Sciences and Comparative Medicine Center, Peking Union Medical College, Beijing, China; 3 Sino Biological Inc., Beijing, China; 4 Comprehensive AIDS Research Center, School of Medicine, Tsinghua University, Beijing, China; Massachusetts General Hospital, United States of America

## Abstract

**Background:**

Pandemic influenza represents a major threat to global health. Vaccination is the most economic and effective strategy to control influenza pandemic. Conventional vaccine approach, despite being effective, has a number of major deficiencies including limited range of protection, total dependence on embryonated eggs for production, and time consuming for vaccine production. There is an urgent need to develop novel vaccine strategies to overcome these deficiencies.

**Methodology/Principal Findings:**

The major objective of this work was to develop a novel vaccine strategy combining recombinant haemagglutinin (HA) protein and a master cell (MC) activator C48/80 for intranasal immunization. We demonstrated in BALB/c mice that MC activator C48/80 had strong adjuvant activity when co-administered with recombinant HA protein intranasally. Vaccination with C48/80 significantly increased the serum IgG and mucosal surface IgA antibody responses against HA protein. Such increases correlated with stronger and durable neutralizing antibody activities, offering protection to vaccinated animals from disease progression after challenge with lethal dose of A/California/04/2009 live virus. Furthermore, protected animals demonstrated significant reduction in lung virus titers, minimal structural alteration in lung tissues as well as higher and balanced production of Th1 and Th2 cytokines in the stimulated splenocytes when compared to those without C48/80.

**Conclusions/Significance:**

The present study demonstrates that the novel vaccine approach of combining recombinant HA and mucosal adjuvant C48/80 is safe and effective in eliciting protective immunity in mice. Future studies on the mechanism of action of C48/80 and potential combination with other vaccine strategies such as prime and boost approach may help to induce even more potent and broad immune responses against viruses from various clades.

## Introduction

Pandemic influenza represents a major threat to global health as novel pathogenic strains can potentially emerge and spread among humans anytime and anywhere around the world. In April 2009, while many of the experts worried about cross-species as well as human to human transmission of the highly pathogenic avian influenza A (HPAI) H5N1 in Asia, the outbreak of a novel strain of H1N1 influenza A virus emerged in Mexico instead. This novel strain, also referred to as “swine flu”, is the recombinant of a previous triple reassortment of bird, pig, and human influenza A viruses that has further recombined with a Eurasian pig influenza A virus [1], [2]. Unlike most strains of influenza A, the pandemic H1N1 does not disproportionately infect adults older than 60 years. In fact, people in this age group showed some trend in resisting infection or had a slower rate of disease progression [3], [4], [5]. A fraction of young and healthy persons, however, developed severe acute respiratory distress syndrome. Furthermore, this novel H1N1 strain demonstrated exceedingly high transmission efficiency among humans throughout the world. Thus, by June 11, 2009, the World Health Organization declared the outbreak of the novel H1N1 to be a pandemic.

Vaccination is the most economic and effective strategy to control influenza pandemic. Conventional influenza vaccines use inactivated whole-virus or disrupted viral antigens, and function by inducing neutralizing antibodies against the highly variable surface glycoproteins, hemagglutinin (HA) and neuraminidase (NA). In general, these vaccines are highly effective in protecting humans from infection and significantly reduce the symptoms of disease [6]. However, a number of major deficiencies are clearly recognized with the current vaccines. First, the breadth of immunity is rather limited and its protective functions are only in the context of infecting viral strains that closely resemble the antigenic specificities of those in the vaccines. As antigenic drift frequently occurs for the virus, annual surveillance and production of new vaccines is required. Second, the production of vaccines is entirely dependent on embryonated eggs. This approach, developed more than half century ago, is time-consuming and can potentially be handicapped by insufficient supplies of eggs which could happen in particular when epidemic of HPAI occurred. The vaccine normally takes at least 6 months to produce and distribute once a potential pandemic strain has been identified. This is exemplified by the vaccine against 2009 H1N1 “swine flu” which was not made available until August, approximately half a year after the identification of the novel virus [7], [8], [9], [10], [11]. In the mean time, the virus continued to spread around the world. It could be imagined that if the virus was deadly, the consequences would be disastrous. Furthermore, the egg-based approach, at least in theory, may fail to adapt the virus to produce sufficient quantities for mass use. During adaption process, the immunogenicity of the viral epitopes could also be altered so that the antibodies induced by them may be different from those against non-culture wild-type strains. Taken together, all these provide a strong argument for the urgent need to develop alternative vaccine strategies to overcome the major deficiencies associated with the conventional vaccine approach.

In June 17, 2003, the US Food and Drug Administration (FDA) approved a novel vaccine for influenza called FluMist, which is the first and the only live attenuated vaccine for influenza available outside of Europe then [12], [13]. The idea for this novel vaccine is at least two-fold. First, as influenza virus infects the upper respiratory tract and cause a contagious acute respiratory disease, vaccine should at least generate sufficient immune responses at the mucosal surfaces to provide the first line of defense against the initial viral attack. Second, the needle-free approach involving nasal spray provides a much easier way for immunization and hence increases the access to the general public. While this novel approach has several obvious advantages over the conventional approaches, it also faces the same drawbacks similar to all of the other live attenuated vaccines. The major ones include its nature of instability which may result in mutant back to the pandemic strain, and potential risk of development of novel chimeric strains between the vaccine and wild-type viruses.

The aim of this study is to develop a safe and effective influenza vaccine which can overcome the shortcomings and, to improve on the strength of above-mentioned vaccine approaches at the same time. In particular, we focus on the development of recombinant HA protein-based vaccine in conjunction with a novel adjuvant (C48/80) targeting mast cells (MC) at the mucosal surface in the nostrils. Compound 48/80 (C48/80) is a polymer that has long been known for its role in activating MC, and so named MC activator [14], [15], [16]. Its role as an effective adjuvant, however, was only identified recently in the context of protection against anthrax lethal toxin challenge as well as vaccinia virus infection *in vivo* [17]. It has also been shown that immunized wild-type mice with C48/80 mixed with a low dose of protective antigen (PA) from Bacillus anthracis resulted in a robust increase in IgG antibody [17]. Such response seems to operate through the activation of dendritic cells (DCs) and mast-cell derived TNF [17], stored in and then released from the granules to mobilize lymphocytes and DCs [17], [18]. The working hypothesis for MC-activator as an adjuvant is that the activation-induced release of inflammatory mediators can lead to activation of DCs containing the immunized antigens and further migration to the draining lymph nodes, thereby reinforcing the antigen-specific immune responses [17], [18], [19], [20], [21].

In this report, we present evidence that 2009 H1N1 HA-based subunit vaccine together with C48/80, when used to immunize mice through the intranasal route, evoked significant increases in HA-specific serum IgG as well mucosal IgA responses in the nostrils, lung, vagina and gut. Such increases were for both binding activities to HA proteins as well as neutralizing activities against live 2009 H1N1 virus. In contrast, no significant increases in serum total and HA-specific IgE was found. Furthermore, this vaccine approach elicited protective immunity against challenge with lethal dose of 2009 pandemic H1N1 influenza. Lastly, the antibody responses induced through our novel approach was comparable in magnitude and duration to that induced by cholera toxin, which has been considered to be a gold standard for mucosal adjuvants. Collectively, our findings indicate that the novel vaccine approach of combining recombinant HA and mucosal adjuvant C48/80 is safe and effective in eliciting protective immunity in mice. It offers the type of influenza vaccine that we urgently need with unprecedented speed and efficacy. The implication of our findings for future vaccine development in humans is also discussed.

## Materials and Methods

### Reagents

The extracellular domain of H1N1 (A/California/04/2009) HA protein containing a His-tag at the C-terminal was produced in 293 cells and purified through affinity chromatograph (Sino Biological Inc. Beijing, China) The protein, comprising 523 amino acid residues, was secreted from the transfected cells with a predicted molecular mass of 59 kDa. As a result of glycosylation, it migrated as an approximately 75–80 kDa band on SDS-PAGE under reducing conditions (data not shown). Cholera toxin (CT) and compound 48/80 (C48/80) were purchased from Sigma-Aldrich (Beijing, China).

### Ethics Statement, immunization and challenge

**Figure 1:**
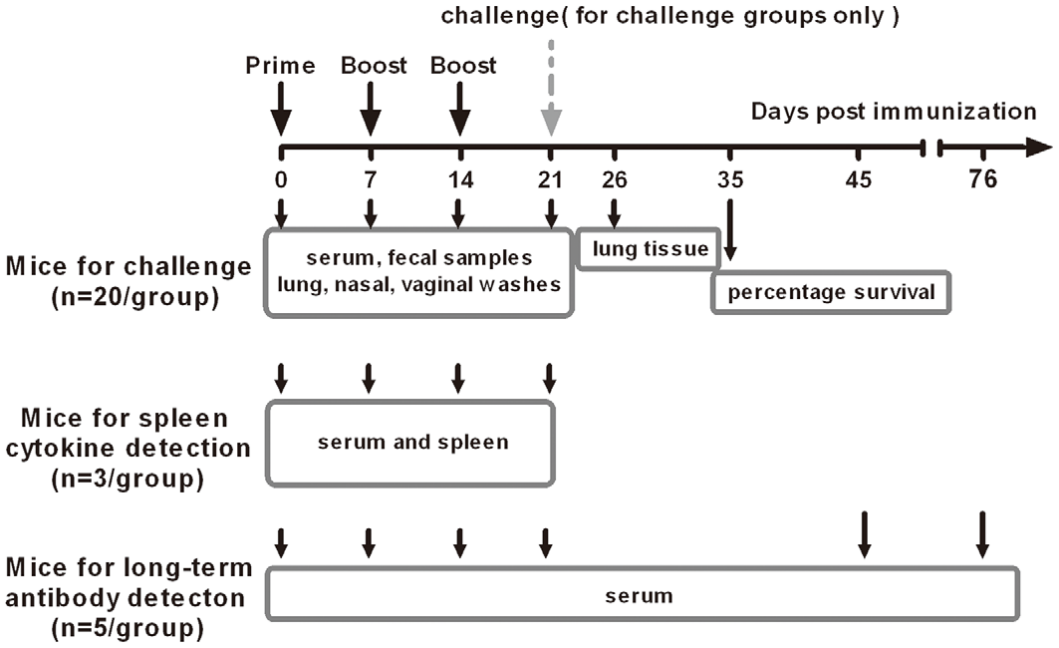
Immunization and sample collection scheme. Four groups of mice, each consisted of 28, were immunized with either PBS, recombinant HA alone, HA plus C48/80 (10 µg) or CT (0.1 µg). Twenty mice were used for challenge with lethal dose of A/California/04/2009 live virus on 21 days after initial immunization and monitored for diseases (n = 10) and viral and pathological investigation in lung tissues (n = 10). The remaining eight were used for either cytokine production from stimulated spleenocytes (n = 3) or long-term antibody response (n = 5).

All procedures for animal use and care were approved by the Institutional Committee on Laboratory Animals at CAMS/PUMC and Tsinghua University (2010-ZhangLQ-ADV). Four-week-old female BALB/c mice were purchased form Institute of Laboratory Animal Sciences at Chinese Academy of Medical Science (CAMS) and Peking Union Medical College (PUMC). Four groups of mice, each consisted of 28 (Fig. 1), were anesthetized with isoflurane followed by intranasal immunization with either PBS for negative control, 9 µg recombinant HA alone or HA plus adjuvants C48/80 (10 µg) or CT (0.1 µg) in a total volume of 15 µl (7.5 µl per nostril). The final dilutions for HA or HA plus adjuvant were made in saline. The immunization scheme and sample collection are as shown in Fig. 1. All mice were primed on day 0, and then boosted on day 7 and 14 days afterwards. Twenty mice from each group were challenged intranasally one week after the second boost (21 days after immunization) with 1×10^6^ TCID_50_ A/California/04/2009 live virus. The virus was a mouse-adapted strain with one amino acid change and became lethal on mice [22], [23]. The virus was initially propagated in 10-day-old embroynated hens' eggs and titered in Madin-Darby canine kidney (MDCK) as previously described [24]. Ten mice in each challenged group were monitored daily for signs of diseases, weight loss, and mortality up to 14 days post challenge. The other ten mice were euthanized at 5 day post challenge and whole lung tissue samples were collected separately for viral nucleic acid quantification and pathological investigation as previously described [22], [23]. Three mice from each group were used to collect spleen samples for cytokine profiling (see below) right before intranasal inoculation of live virus challenge. Five mice from each group were used to monitor the long-term antibody responses against HA for up to day 76 after first immunization (Fig. 1).

Serum, vaginal and fecal samples were collected on day 0, 7, 14 and 21. Lung and nasal washes were collected on day 21. Serum collections were also done on day 45 and 76 for monitoring antibody responses (Fig. 1). All mice were anesthetized and blood samples were collected from mice tail into 1.5 ml centrifuge tubes by vein puncture. After clotting overnight at 4°C, the blood was centrifuged at 2500 rpm for 30 min. The serum was transferred to a new 1.5 ml centrifuge tube and stored at −80°C until time of testing. A total of 100 µl PBS was used for vaginal washing. PBS stayed at least 10 sec in the mouse vagina for each washing and was washed at least 3 times for each mouse. Vaginal lavage sample was centrifuged at 13,000 rpm for 30 min, the supernatant was transferred to a new 1.5 ml centrifuge tube and stored at −80°C. Fresh fecal sample from each mice were also collected into a 1.5 ml centrifuge tube with 100 µl PBS and were vortexed severely until thoroughly mixed. The mixture was centrifuged at 13,000 rpm for 30 min and the supernatant was transferred into a new 1.5 ml centrifuge tube and stored at −80°C. Mice lung and nasal washes were collected as previously described [25]. For lung washes, mice trachea and lungs were removed after euthanized, and were washed 3 times by 2 ml of PBS containing 0.1% BSA. For nasal washes, mice were decapitated and lower jaws were excised. 1 ml of PBS containing 0.1% BSA was injected into the posterior opening of the nasopharynx of mice 3 times to collect the nasal washes. All washes were centrifuged to removing cellular debris and the supernatant were stored at −80°C for later use.

### Histology

For pathological examination, lung samples were fixed in 10% formalin, embedded in paraffin and sectioned. Conventional hematoxylin-eosin (H&E) staining was conducted on serial 4 µm tissue sections and examined under the light microscope by a pathologist blinded to the experimental groups.

### Measurement of binding antibodies against HA

Binding antibody was measured using conventional ELISA technique. In brief, 96-well plates were coated with 100 µl/well of H1N1 HA protein at 1 µg/ml in carbonate coating buffer overnight at 4°C. After blocking with 10% FBS and 0.05%Tween in PBS, the plates were washed twice with 0.05% Tween in PBS. Serially diluted serum, nasal and lung washes, and vaginal and fecal samples in PBS, were added at 100 µl per well and incubated for 1 hour at 37°C. After 3 washes, horseradish peroxidase (HRP)-conjugated goat anti-mouse IgG antibodies (Promega, US), anti-IgA antibodies and anti-subclass IgG1, IgG2a, IgG2b, IgG3 antibodies (Santa Cruz, US) were added and incubated at 37°C for another hour. After extensive washes, 50 µl of HRP-substrate 3,3′,5,5′-tetramethylbenzidine (TMB) was added, incubated for 5 minutes, and stopped by adding 50 µl 1 M H_2_SO_4_. Samples were measured at an absorbance of 450 nm. End-point titer were recorded as the reciprocal log_2_ dilution of the last sample whose OD was at least 3-fold higher than that of the corresponding naive sample.

### Measurement of total and HA-specific IgE in immunized mice

Mouse serum total IgE were detected by Mouse IgE ELISA Set (BD Biosciences, USA). Briefly, 96-well plates were coated with 100 µl/well of capture antibody diluted in coating buffer ( 0.1 M Sodium Carbonate, PH 9.5 ) at 1∶250 dilution and incubated overnight at 4°C. After blocking with assay diluent (PBS with 10% FBS ), serum samples and IgE standard were added at 100 µl/well and kept in 37°C for 2 hours. IgE standard was prepared at two-fold dilutions in assay diluents starting at 50 ng/ml. Following incubation, plates were washed and detection antibody (1∶1000) with enzyme reagent (1∶250) diluted in assay diluents were added at 100 µl/well. After incubation at 37°C for 1 hour, 50 µl substrate TMB was added, incubated for 5 minutes, and stopped by adding 50 µl 1 M H_2_SO_4_. The plate was read at 450 nm and OD values were compared to the standard curve to determine the IgE concentration of each serum. The method for determining antigen-specific IgE titers was similar to the total IgE ELISA except that the coating protein was H1N1 HA at 1 µg/ml in carbonate coating buffer.

### Measurement of neutralizing antibodies against A/California/04/2009 live virus

Neutralizing antibodies were measured by using a neutralization assay based on MDCK cell culture. In brief, MDCK cells were seeded at 1.5×10^4^ cells/well in 96-well culture plates and incubated overnight at 37°C. All mouse serum samples were inactivated at 56°C for 45 min. Serial two-fold dilutions of each serum samples were mixed separately with 100 TCID_50_ of A/California/04/2009 live virus and incubated at 37°C for 2 h. The mixture was then added directly to MDCK cells in the 96-well culture plates, and the neutralizing titers of mouse serum were assessed based on presence or absence of cytopathic effect (CPE) 3 days post infection. Neutralization antibodies titers were determined as the reciprocal of the serum dilution that decreased by 50% the number of CPE wells formed by the live virus and the 50 percent endpoints were computed by Reed-Muench method [26].

### Viral load measurement in lung by real-time PCR

Total RNA was isolated from mice lung tissues by using the RNeasy Mini Kit (Qiagen, US). RNA was dissolved in 30 µl diethyl pyrocarbonate-treated water and stored at −80°C. First-strand cDNA was synthesized by using random hexamers containing 200 Unit of Superscript III reverse transcriptase (Invitrogen, US). The real-time relative quantitative PCR assays were performed on ABI PRISM®7500 in a total of 20 µl volume containing 2 µl cDNA, CSqPCR Master Mix (Shanghai Chaoshi Bio Technologies Co., Ltd.), pair primers and specific Taqman probe for either H1N1 or mouse GAPDH transcripts. The sequences for primers and probes are list as follows: H1N1-F:5′-GTG CTA TAA ACA CCA GCC TYC CA -3′; H1N1-R:5′-CGG GAT ATT CCT TAA TCC TGT RGC -3′ H1N1-Probe: 5′-FAM-CA GAA TAT ACA TCC RGT CAC AAT TGG ARA A-BHQ-3′; GAPDH-F:5′-GCA CAG TCA AGG CCG AGA A-3′, GAPDH-R:5′-CCT CAC CCC ATT TGA TGT TAG TG-3′, GAPDH-Probe: 5′-FAM-CATC ACC ATC TTC CAG GAG CGA GAC C-BHQ1-3′. Thermal cycling was conducted under the following conditions: 95°C for 10 min, followed by 40 cycles of 95°C for 30s and 60°C for 40s. Lung relative viral load was normalized to H1N1 RNA loading for each sample using the GAPDH RNA as an internal standard. The relative quantity of lung H1N1 virus RNA was given as 2^−ΔΔCT^. ΔΔCT was calculated as follows: ΔΔCT = ΔCT(target)−ΔCT(reference) [27].

### Measurement of cytokine release by stimulated splenocytes in mice

Three mice from each group were used to study cytokine profiles from stimulated splenocytes. Mice were euthanized one week after the second boost (21 days after immunization), coinciding with the time right before virus challenge (see above). The spleens were immediately collected and made into single cell suspension. Cells were washed three times and plated in 48 well culture plates at 2.5×10^6^ cells/well with RPMI 1640 medium , 10% heat-inactivated fetal bovine serum (FBS), and 1% penicillin and streptomycin. Five micrograms (5 µg) of H1N1 HA protein was then added to each well to yield a final concentration of 5 µg/ml. These cell culture plates were incubated in 5% CO_2_ incubator at 37°C and supernatants were harvested 72 hours later. Collected supernatant was first centrifuged to remove cell debris and then stored at −80°C until use. The cytokines in the supernatant including IL-2, IL-4, IL-6, IL-17, TNF and IFN-alpha were determined by flow cytometry using a mouse Th1/Th2/Th17 CBA Kit (BD Biosciences, US).

### Statistic analysis

We used unpaired two sample t-test to compare serum IgG, lung, nasal washes, vaginal and fecal IgA , as well as relative viral load in lung tissue after challenge between groups. A value of p≤0.05 was considered statistically significant. ANOVAs for multiple comparisons were performed for comparison of parameters between groups for serum IgG subclasses. All statistic analysis was performed using GraphPad Prism 5 (GraphPad Software Inc., San Diego CA, www.graphpad.com.).

## Results

### MC activator C48/80 significantly increases systemic and mucosal antibody responses against HA protein through intranasal immunization

**Figure 2:**
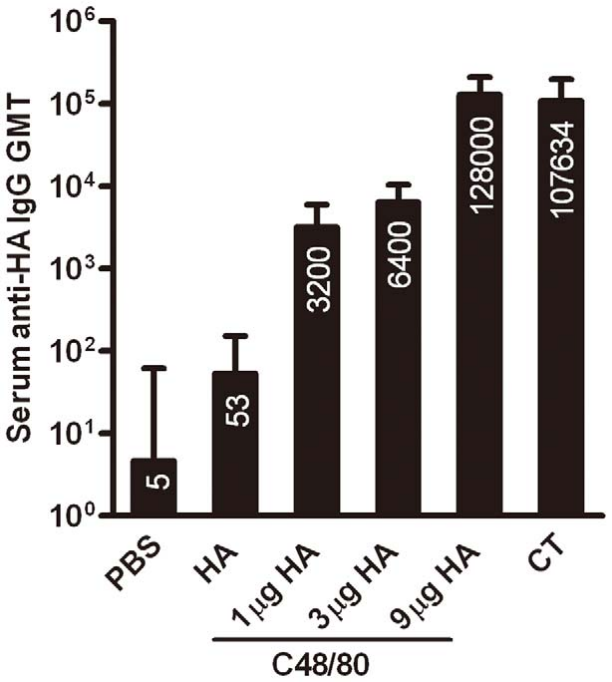
Geometric mean titers (GMT) for serum anti-HA antibody responses after immunization with three doses (1 µg, 3 µg, or 9 µg) of recombinant HA together with C48/80, and compared with HA alone (9 µg) or plus CT. The numbers on the bar indicate the actual value of GMT.

To investigate whether C48/80 could be used as an adjuvant for influenza recombinant HA protein, we first immunized 4 mice each with 1 µg, 3 µg, or 9 µg recombinant HA coupled with 10 µg C48/80 on day 0, 7 and 14 through intranasal route. Fig. 2 demonstrates the geometric mean titers (GMT) of serum IgG binding antibodies against HA protein for the 3 dose groups on day 21. It is clear that recombinant HA protein (9 µg) alone induced detectable but borderline serum binding antibody with GMT of 53. However, the same amount of HA protein administered together with C48/80 induced significantly higher levels (P<0.05) of binding antibody with GMT reaching 128,000, which was more than 3 logs higher compared to that with HA alone. In addition, as the dose of HA decreased from 9 ug to 3 µg and 1 µg , there was corresponding decline in binding antibody with GMT at 6,400 and 3,200 respectively. Furthermore, levels of binding antibody in the 9 ug HA plus C48/80 group (128,000) was comparable to that in the 9 ug HA plus CT group (107,634). Lasting, we also measured the total IgE and HA-specific IgE antibody concentration in the immunized mice. No significant differences were found in total IgE between mice immunized with HA alone (76.36±43.78 ng/ml) or with C48/80 (83.99±15.02 ng/ml) or CT (66.58±31.24 ng/ml) (Table 1). Dose differences in recombinant HA did not resulted in discernable differences in total IgE concentration. In particular, no HA-specific IgE was detected in any of the immunized group, suggesting that MC activator C48/80 did not induce IgE production or IgE-associated allergic and hypersensitivity reactions. We have therefore chosen the 9 ug HA group for more detailed immunologic characterization and challenges experiment with A/California/04/2009 live virus.

**Table 1:** Concentration of serum total IgE (n = 4/group).

Group	Serum total IgE (ng/ml)
PBS	37.06±22.25
HA 9 µg	76.36±43.78
HA 1 µg+c48/80 10 µg	78.27±38.9
HA 3 µg+c48/80 10 µg	104.1±41.45
HA 9 µg+c48/80 10 µg	83.99±15.02
HA 9 µg+CT 0.1 µg	66.58±31.24

**Figure 3:**
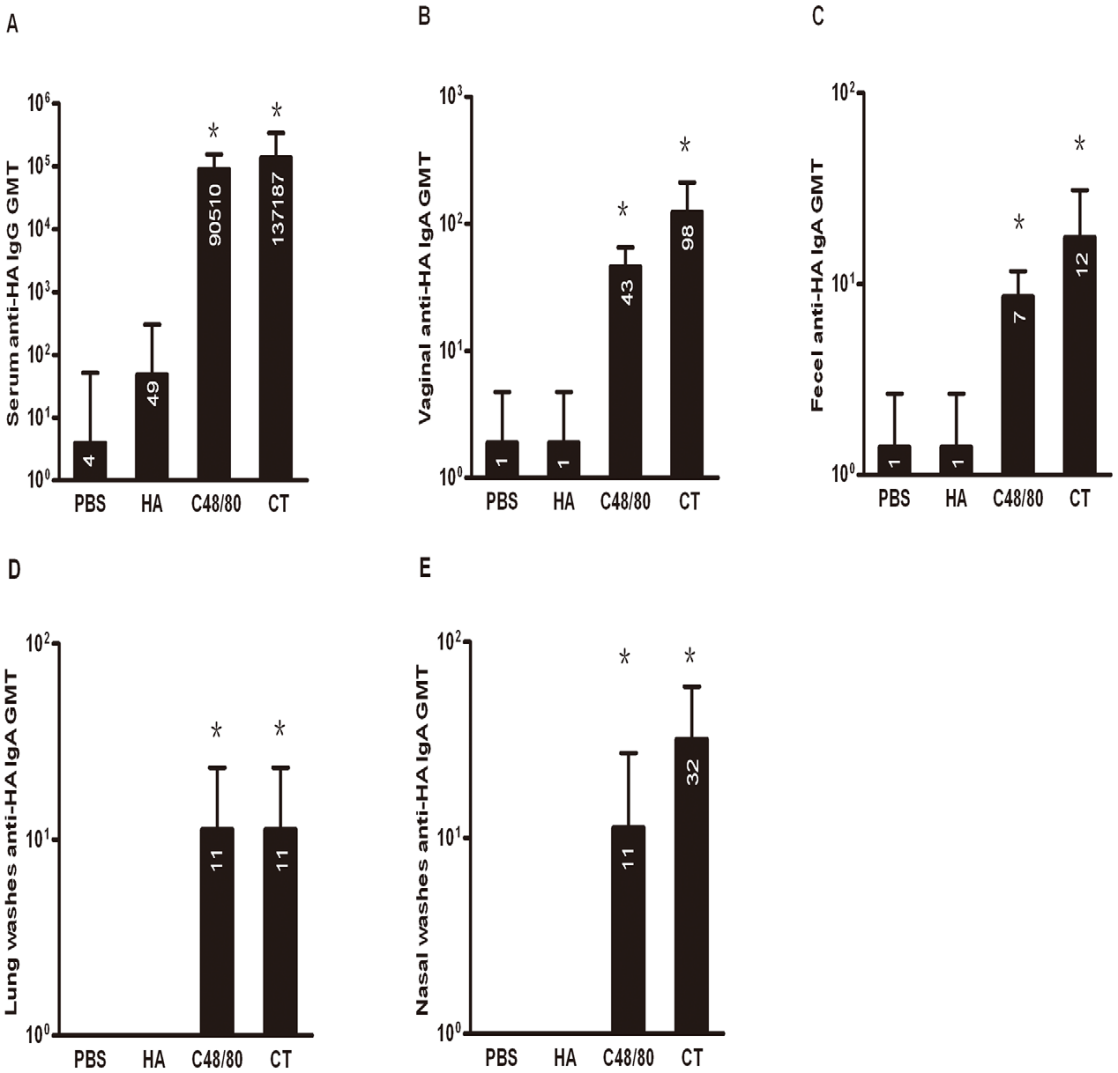
Geometric mean titers (GMT) for serum (A) and mucosal (B: vaginal; C: fecal; D: lung; and E: nasal) anti-HA antibody responses of four groups of animals (n = 4–10 per group) measured on 21 days after initial immunization by ELISA. Bars represent the GMT of all animals in each group with error bars representing the 95% confidence. The numbers on the bar indicate the actual value of GMT. Star (*) indicates the significant difference in antibody titers between HA alone and HA plus C48/80 or plus CT (p<0.05).

We immunized 28 mice each with either 9 µg recombinant HA alone, with C48/80 (10 µg), with PBS or CT (0.1 µg) in a total volume of 15 µl (7.5 µl per nostril) on day 0, 7, and 14 (Fig. 1). Fig. 3A demonstrates GMT of serum IgG binding antibodies against HA protein among the four groups one week after the final immunization (day 21 after initial immunization). Recombinant HA protein alone induced detectable but borderline serum binding antibody with GMT of 49. However, the same amount of HA protein administered together with C48/80 induced significantly higher levels (P<0.05) of binding antibody with GMT reaching 90,510, which was more than 3 logs higher compared to that with HA alone. Furthermore, levels of binding antibody in the HA plus C48/80 group was comparable to that in the HA plus CT group (13,7187), suggesting MC activator C48/80 had similar potent adjuvant effect at the mucosal surface as that of CT, which is considered to be a gold standard for mucosal adjuvants. The GMT for PBS only group was about 4 (Fig. 3A).

In addition, vaginal lavage and fecal extracts as well as lung and nasal washes from the four groups were also tested for HA-specific IgA binding antibody one week after the final immunization (day 21 after initial immunization). Fig. 3B,3C, 3D, and 3E show that, similar to IgG binding antibodies, intranasal immunization with HA protein plus C48/80 induced significantly higher levels (P<0.05) of IgA binding antibody compared to that with HA alone at the vaginal and gastrointestinal as well as nasal and lung surfaces. The GMT for the HA plus C48/80 group was 43 for vaginal lavage (Fig. 3B),7 for fecal extracts (Fig. 3C), 11 for lung and nasal washes (Fig. 3D and 3E), while that for the HA alone and negative control group were under the detection limit. Furthermore, such levels of IgA antibody responses were equivalent to that induced by CT which was 98 for vaginal lavage,12 for fecal extracts, 11 for lung washes (Fig. 3D) and 32 for nasal washes (Fig. 3E). These findings indicate that MC activator C48/80 can significantly increase the immunogenicity of HA protein both systemically and at the mucosal surfaces, and its adjuvant effect is comparable to that of CT.

### MC activator C48/80 significantly increases the durability of serum antibody responses against HA protein through intranasal immunization

**Figure 4:**
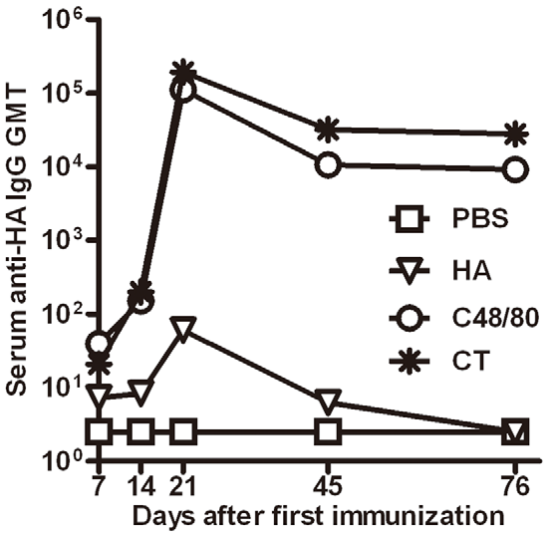
High and durable levels antibody responses in animals immunized with HA plus C48/80 or plus CT compared to those with HA alone or negative control PBS (n = 5 per group).

To investigate the impact of C48/80 on the persistence of antibody responses, we monitored the dynamic changes of serum anti-HA IgG titers over a 76-day period from the initial immunization. As shown in Fig. 4, there are dramatic changes in GMT of serum IgG binding antibodies against HA protein among the four groups over the course of and post immunization. After each immunization, increasing levels of antibody responses were found for animal group immunized with either HA alone, HA plus C48/80, or HA plus CT. However, the rate of increases was more profound for the latter two groups compare to the first one. On day 21, one week after the third immunization, the levels of antibody reached the peak for all three groups but with more than 3 logs difference in antibody GMT between the latter two and the first group (Fig. 4). In particular, when antibody GMT in the HA alone group declined to below the detection limit on day 45 after initial immunization, high levels of antibody were still present in the other two groups despite an approximately one log decline in absolute GMT compared to the peak value (Fig. 4). More importantly, such high levels of antibody responses in the two groups persisted over the following month without appreciable decline, although HA plus CT group (27,857) had about 3 times higher in absolute GMT compared with HA plus C48/80 group (9,190). No changes in antibody levels were identified for negative control animals throughout the study period. These findings suggest that MC activator C48/80 can induce durable antibody responses when used as an adjuvant against HA recombinant protein through intranasal immunization.

### High levels of binding antibodies against HA protein boosted by C48/80 corresponds to the high levels of neutralizing antibodies against A/California/04/2009 live virus

**Figure 5:**
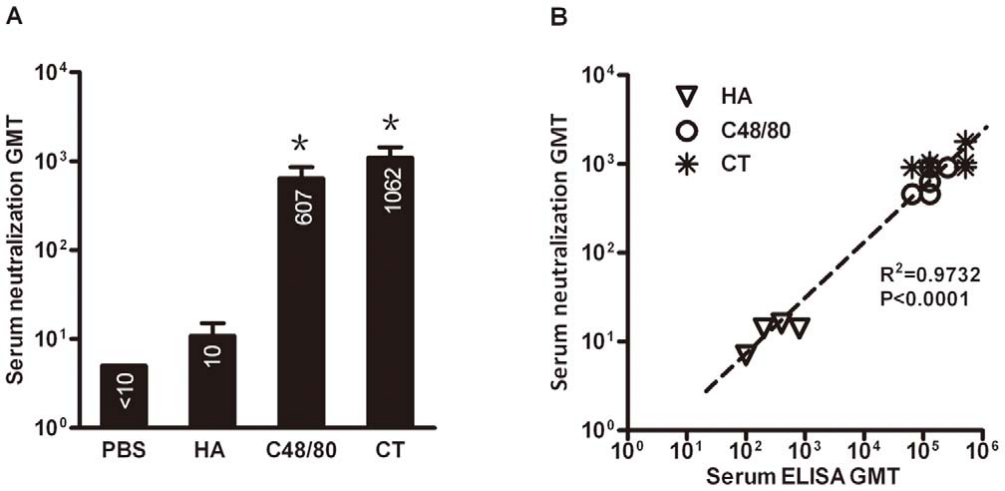
(A) Comparison of serum neutralizing antibody activities against A/California/04/2009 live virus among the four groups of animals (n = 6 per group) immunized with PBS, HA alone, HA plus C48/80 or HA plus CT. The numbers on the bar indicate the actual value of GMT. Star (*) indicates the significant difference in neutralization titers between HA alone and HA plus C48/80 or plus CT (p<0.05). (B) Positive correlation between the serum binding and neutralizing antibody activities in all animals tested(n = 6 per group).

Antibody binding activity does not always translate into neutralizing capability. To study the neutralizing potential of serum samples from immunized animals, we performed neutralization assay based on A/California/04/2009 live virus and MDCK cells in vitro. As the antibody binding activity is the highest on day 21 after initial immunization, we used this time point serum samples to evaluate their neutralization activities. Serial two-fold dilutions of each serum samples from all four groups were mixed separately with 100 TCID_50_ of A/California/04/2009 live virus, and the neutralizing titers for each serum sample, hence the GMT for each animal group, were assessed based on presence or absence of cytopathic effect (CPE) 3 days post infection. As shown in Fig. 5A, while HA alone group failed to demonstrate detectable neutralizing activity, co-administration of HA with C48/80 or CT resulted in significant higher levels of neutralizing activity with GMT of 607 for the former and 1062 for the latter. In addition, a significant correlation (P<0.0001) was found between the antibody binding activities and neutralizing capacity, reflected by high correlation coefficient (R^2^) value of 0.9732 using a linear regression model (GraphPad Prism 5) (Fig. 5B). All these results demonstrate that C48/80 can significantly boost binding as well as neutralizing antibodies targeted on HA protein, therefore can potentially be a good candidate for mucosal adjuvant.

### Humoral responses boosted by C48/80 offer protective immunity against lethal challenge by A/California/04/2009 live virus

**Figure 6:**
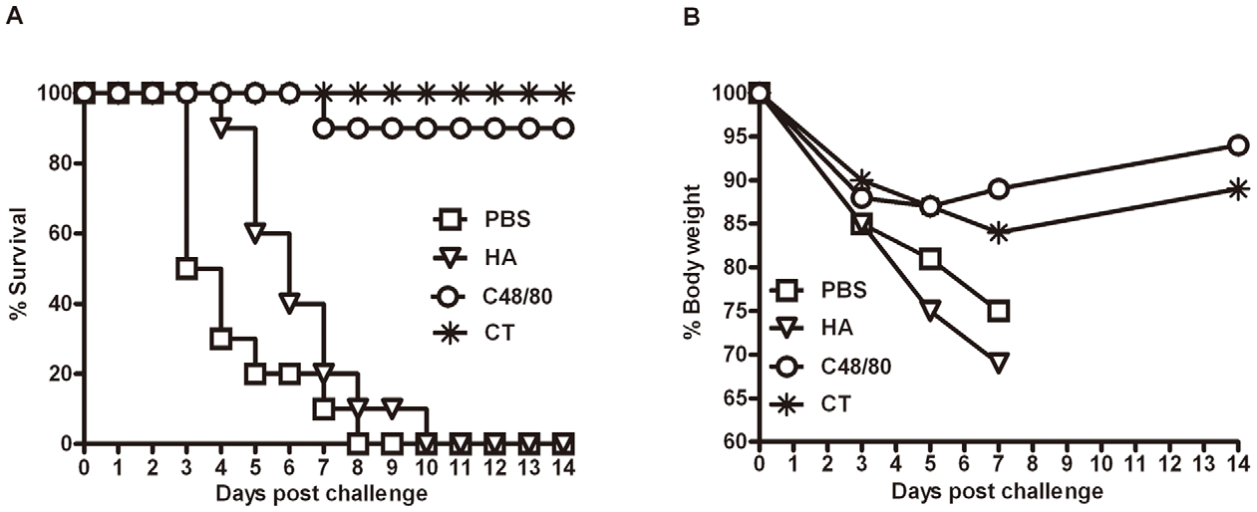
(A) Survival percentage of four groups of animals (n = 10 per group) immunized with PBS, HA alone, HA plus C48/80 or HA plus CT. (B) Body weight changes.

We further studied whether the high levels of antibody responses boosted by C48/80 could provide any protective immunity against lethal virus challenge. Twenty mice from each study group were challenged intranasally with 1×10^6^ TCID_50_ of live A/California/04/2009 on day 21, corresponding to one week after the third immunization (Fig. 1). Of these, ten were scarificed on day 5 after challenge to collect lung tissues for viral RNA measurement and histopathological changes, while the remaining ten were monitored for body weight loss and mortality over a two-week period. The HA plus C48/80 group demonstrated high protection rate with 9 out of 10 animals surviving up to 2 weeks after challenged. No animals in HA plus CT group succumbed to diseases. In contrast, all animals in the HA alone or in the negative control group died 10 days after challenge (Fig. 6A). Furthermore, the percent of survival was highly correlated with the percentage of body weight recovery after challenge. As shown in Fig. 6B, despite initial loss in body weight after challenge, animals in the group that received HA plus C48/80 or plus CT began to recover on day 7 after challenge, while those in HA alone or negative control groups continued to drop their weight until death. These findings collectively suggest that high levels of antibody responses boosted by C48/80 can offer protective immunity against diseases to the level that is equivalent to that of CT.

### Protected animals demonstrate minor histological changes and decreased viral loads in lung tissues

**Figure 7:**
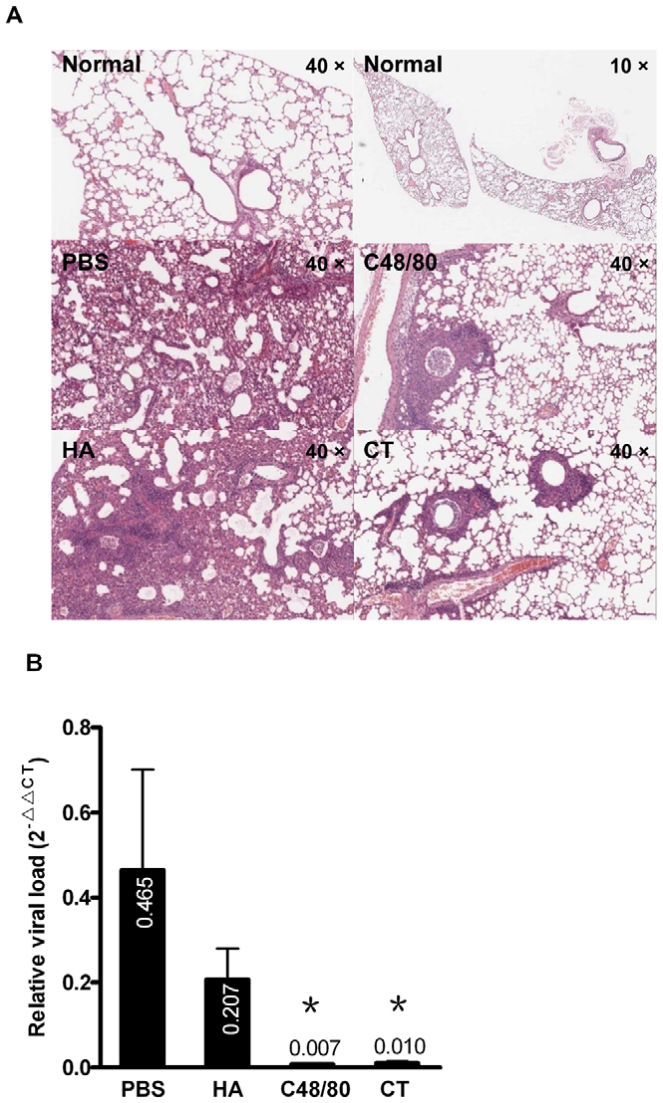
(A) Pathological analysis of the lung tissues from normal animals or those immunized with PBS, HA alone, HA plus C48/80 or HA plus CT after challenge with A/California/04/2009 live virus. (B) Comparison of lung viral RNA between animals (n = 6 per group) immunized with PBS, HA alone, HA plus C48/80 or HA plus CT. The numbers on the bar indicate the actual value of relative viral load. Star (*) indicates the significant difference in viral RNA between HA alone and HA plus C48/80 or plus CT (p<0.05).

To study the virologic and histopathologic changes in immunized animals post challenge, lung tissues were collected on day 5 after challenge and processed for H&E staining. Fig. 7A shows representative sections from the four animal groups. The overall condition from HA plus C48/80 or CT group was quite comparable to that found in the normal animals except for minor degree of lymphocyte infiltration and aggregation around pulmonary capillary as well as bronchiole wall. In contrast, animals in HA alone or the negative control group exhibited severe damage to the lung parenchyma, reflected by massive lymphocyte infiltration, inflammatory hyperaemia, slight hemorrhage, edema, and exudative pathological changes. These findings showed that immunization of HA together with C48/80 or CT protected animals from lung damage after challenge with live A/California/04/2009.

Further experiment was carried out to detect lung relative viral loads by real time RT-PCR. Fig. 7B demonstrates a relative viral load of 0.207 and 0.465 in mice that received HA alone and no vaccination (negative control group) respectively. This translated to 20∼50 times higher viral loads than those in the HA plus C48/80 or CT group. There was no significant difference, however, found between the HA plus C48/80 and plus CT group. The higher survival rates in these two groups were likely due to significant reduced viral replication and replication related inflammation.

### Significant differences in IgG subclass & cytokine production among different animal groups

**Figure 8:**
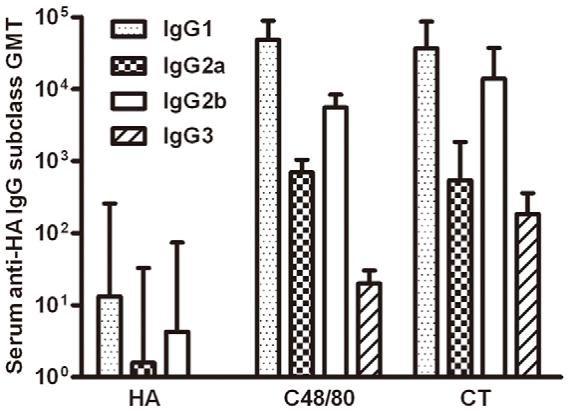
GMT of serum anti-HA IgG1, IgG2a, IgG2b, and IgG3 in animals (n = 10 per group) immunized with HA alone, HA plus C48/80 or HA plus CT on day 21 after initial immunization.

The pattern of IgG subclasses reflects the subset of CD4+ T helper (Th) cells polarized in immune responses leading to the different mechanisms of host protection process [28], [29]. Generally, IgG1 corresponds to Th2-biased responses, while IgG2a corresponds to Th1-biased responses. To further investigate which subset of Th cells was induced by HA plus C48/80 when immunized intranasally, we monitored the GMT of serum HA-specific IgG1, IgG2a, IgG2b and IgG3 in immunized animals on day 21 after initial immunization (Fig. 1). As shown in Fig. 8, HA plus C48/80 or plus CT induced marked increases for all IgG subclasses compared to the HA alone group. Such increases were in the order of 1–3 logs relative to that in the HA alone group (Fig. 8). These findings suggest that C48/80, like that of CT, is able to induce more balanced Th1/Th2 responses, consistent with observed increases in antibody responses in immunized animals. However, IgG1 titers were significantly higher than that of IgG2a, IgG2b and IgG3 in all animal groups suggesting our recombinant HA-based mucosal vaccine strategy, while quite balanced between Th1/Th2, preferentially induces Th2-biased immune responses. Furthermore, IgG2b titer was about 8-fold higher than IgG2a in all immunized group, which was different from earlier report where the reverse was observed when C48/80 was given intradermally [30]. Lastly, the IgG3 levels were relatively low and only became detectable when dilution factor was less than 200 in HA plus C48/80 or plus CT group while that in the HA alone remained undetectable (Fig. 8).

To further investigate the ability of C48/80 in influencing responses induced by HA recombinant vaccine, spleen were harvested on day 21 after initial immunization (Fig. 1) and re-stimulated with HA protein. Supernatants were collected 72 h later and evaluated for various cytokines. As illustrated in Table 2, supernatants from PBS or HA alone group had undetectable levels of cytokines. Supernatant from HA plus C48/80, however, had marked increased levels of IL-2, IL-6, and IL-10, whereas for HA plus CT group, increases were found for all cytokines except for IL-4. As IL-2 is a Th1 cytokine, whereas IL-6 and IL-10 are both Th2 cytokines, the cytokine profile is quite consistent with what is expected, reinforcing the notion that C48/80 can induce balanced Th1/Th2 responses. In particular, IL-6 is critical for the development of follicular helper T (Tfh) cells for the activation and maturation of B cells for antibody production [31], [32]. Lastly, as CT is able to induce high levels of wide array of cytokines, this could provide the reason why CT has such a strong adjuvant effects.

**Table 2:** Antigen-specific cytokine production from splenocytes after stimulation (n = 3/group).

Group	Stimulator	Cytokine production (pg/ml)						
		IL-2	IL-4	IL-6	IFNγ	TNF	IL-17	IL-10
PBS control	None	2±2	2±2	2±2	1±1	1±1	0±2	1±2
PBS control	PHA	8±2	2±2	107±63	95±94	339±79	34±33	25±26
PBS control	HA	11±2	1±2	7±1	1±1	15±4	1±2	3±5
HA alone	HA	9±3	0±0	18±13	0±0	11±10	0±0	0±0
HA+C48/80	HA	49±18	1±1	90±25	3±2	10±7	7±4	22±21
HA+CT	HA	580±211	1±2	152±41	114±39	106±50	661±184	45±30

## Discussion

In this study, we demonstrated that MC activator C48/80 had strong adjuvant activity when co-administered with recombinant HA protein through intranasal route. Vaccination with C48/80 significantly increased the serum IgG and mucosal surface IgA antibody responses against HA protein. Such increases correlated with stronger and durable neutralizing antibody activities, offering protection to vaccinated animals from disease progression after challenge with lethal dose of A/California/04/2009 live virus. Furthermore, protected animals showed significant reduction in lung virus titers, minimal structural alternation in lung tissues as well as higher and balanced production of Th1 and Th2 cytokines in the stimulated splenocytes compared to those without C48/80.

This study is the first to demonstrate that intranasal vaccination with recombinant HA protein plus MC activator C48/80 is able to induce protective immunity in mice. Although the dose and immunization schedule need to be further optimized, especially for human use, our results have provided the proof of principle for the safety and efficacy of this novel vaccine strategy and its potential use to overcome the short comings associated with conventional influenza vaccine strategies [33]. In particular, using recombinant HA protein instead of inactivated or attenuated viruses as immunogen will eliminate the total dependence on embryonated eggs and significantly shorten the time required for vaccine production, as the production technology for recombinant protein is becoming more standard and efficient. At the same time, it will also avoid potential adverse effect associated with egg-based products such as hypersensitivity reactions and Guillain-Barré syndrome (GBS), a rare peripheral neuropathy leading to paralysis and in severe cases respiratory failure and death. In addition, as the C48/80 can significantly boost the immunogenicity of recombinant HA protein through intranasal route, such immunization strategy can provide protective immunity at the entry point of natural influenza infection and offers a needle-free convenience compared to injection-based approach, especially when mass immunization is needed.

Our results have also confirmed the observations of others that intranasal immunization is an effective and feasible immunization strategy [34], [35], [36]. Among the mucosal adjuvants being studied, *Vibrio cholerae* cholera toxin (CT) and Escherichia coli heat-labile enterotoxin (LT) are among the most potent known to date [37], [38], [39]. However, given their intrinsic toxic effect, neither of them can be used for human vaccine in their native form. Despite many attempts to improve the safety profile while maintaining its mucosal adjuvant activity [40], [41], [42], [43], a recent human phase I trial with a mutant LT against human immunodeficiency virus (HIV) and tuberculosis shown a transient facial nerve paralysis in two trial participants, raising a serious concern on the safety profile of this class of adjuvant and its future use in human vaccine [44]. In contrast, MC activator C48/80 demonstrated much safer profile in animals while showing comparable adjuvant activity with CT [17], [30], suggesting C48/80 may have potential to become a new and lead class of mucosal adjuvant for human use. This hypothesis is supported by previous reports where C48/80 has been shown to provide adjuvant activity to anthrax recombinant antigens immunized either through intranasal or intradermal route without apparent adverse effects [17], [30]. Although the underlying mechanism of C48/80's adjuvant effect is not entirely clear, it has been hypothesized that it can trigger mast cells to rapidly release inflammatory mediators such as TNF, which in turn induce DCs migration to draining lymph nodes, thereby boosting antigen-specific responses [17], [18], [19], [20], [21]. In fact, recent studies also suggest that mast cells can potentially facilitate the development of adaptive immunity through its effector functions and/or by releasing multiple cytokines, chemokines and growth factors through various mechanisms [20], [21], [45], [46]. Future studies, however, will be needed to delineate the mechanism of action and the specificity of C48/80 in order to further develop it into a safe and effective mucosal adjuvant for human use.

In summary, our findings indicate that the novel vaccine approach combining recombinant HA and mucosal adjuvant C48/80 is safe and effective in eliciting protective immunity in mice. It is a step closer to the ultimate success of recombinant influenza vaccine, offering high speed and efficacy over the conventional strategies. Future studies on the mechanism of action of C48/80 and potential combination with other vaccine strategies such as prime and boost approach may help to induce even more potent and broader immune responses against viruses from different groups.
